# Anti-Staphylococcal Activity of *Ligilactobacillus animalis* SWLA-1 and Its Supernatant against Multidrug-Resistant *Staphylococcus pseudintermedius* in Novel Rat Model of Acute Osteomyelitis

**DOI:** 10.3390/antibiotics12091444

**Published:** 2023-09-13

**Authors:** Sung-Yong Park, Hong-Jae Lee, Hyo-Sung Kim, Dong-Hwi Kim, Sang-Won Lee, Hun-Young Yoon

**Affiliations:** 1 Laboratory of Veterinary Surgery, College of Veterinary Medicine, Konkuk University, Neungdong-ro 120, Seoul 05029, Republic of Korea; ullabunge@konkuk.ac.kr; 2 Laboratory of Infectious Diseases and Veterinary Microbiology, College of Veterinary Medicine, Konkuk University, Neungdong-ro 120, Seoul 05029, Republic of Korea; ihj90@konkuk.ac.kr (H.-J.L.); opeean0@naver.com (D.-H.K.); odssey@konkuk.ac.kr (S.-W.L.); 3 Laboratory of Veterinary Clinical Pathology, College of Veterinary Medicine, Konkuk University, Neungdong-ro 120, Seoul 05029, Republic of Korea; w6373515@naver.com

**Keywords:** osteomyelitis, *Staphylococcus pseudintermedius*, multidrug resistance, antibiotic alternatives, antibacterial activity, antimicrobial compounds

## Abstract

Osteomyelitis caused by staphylococcal infection is a serious complication of orthopedic surgery. *Staphylococcus pseudintermedius* is the main causative agent of osteomyelitis in veterinary medicine. Methicillin-resistant *S. pseudintermedius* (MRSP) has been reported in companion animals, especially dogs. Multidrug-resistant *S. pseudintermedius* is an emerging pathogen and has acquired antibiotic resistance against various commercial antimicrobial agents. New antimicrobial compounds are urgently needed to address antibiotic resistance, and the development of novel agents has become an international research hotspot in recent decades. Antimicrobial compounds derived from probiotics, such as bacteriocins, are promising alternatives to classical antibiotics. In this study, the antibacterial activities of *Ligilactobacillus animalis* SWLA-1 and its concentrated cell-free supernatant (CCFS) were evaluated in vitro and in vivo. The CCFS of this bacterium showed no toxicity against osteoblast and myoblast cells in vitro, while significantly inhibiting the multidrug-resistant *S. pseudintermedius* KUVM1701GC strain in a newly established rat model. The CCFS significantly inhibited multidrug-resistant staphylococci both in vitro and in vivo. This suggests that CCFS derived from *L. animalis* SWLA-1 has potential as an alternative to classic antibiotics for staphylococcal infections in dogs.

## 1. Introduction

Infectious osteomyelitis caused by microorganisms is a serious complication of human and veterinary orthopedic surgeries. Currently, treatment options for infectious osteomyelitis include implant removal, lavage of the surgical site, long-term systemic antimicrobial therapy, revision surgeries, and radical debridement [1,2]. However, these options have disadvantages including the systemic side effects of antimicrobials, drug resistance, and high financial costs [2,3]. Despite efforts to perform surgeries under aseptic conditions and the use of prophylactic antibiotic treatments, implant-related infections remain a challenge [4].

*Staphylococcus* spp. are the predominant bacteria responsible for infectious osteomyelitis. These bacteria are mainly transmitted through opportunistic infections and have various mechanisms of antimicrobial resistance. Methicillin-resistant *Staphylococcus pseudintermedius* (MRSP) has become a major concern in both human and veterinary medicine [5,6]. In small animals, *Staphylococcus pseudintermedius* has been identified as the primary pathogen responsible for infectious osteomyelitis [7]. The recent emergence of methicillin-resistant *S. pseudintermedius* and multidrug-resistant (MDR) *S. pseudintermedius* has posed a significant challenge to veterinary practice [8,9]

To tackle the antibiotic resistance of pathogenic bacteria and secure important antimicrobial agents in human and veterinary medicine, many organizations are dedicated to reducing antibiotic usage [10,11]. In addition, several alternatives to classical antibiotics have been suggested by numerous studies, such as bacteriophage-based therapies, natural probiotic-derived antimicrobial compounds, and nanoparticles [12,13,14,15]. Among them, antimicrobial molecules produced by probiotics, including bacteriocins and organic acids, are promising alternatives to classical antibiotics for fighting MDR bacteria. Various types of these molecules have been discovered and reported to have antimicrobial effects in previous studies [16,17,18,19].

*Ligilactobacillus animalis*, formerly called *Lactobacillus animalis*, is one of the predominant bacteria in the canine fecal microbiota. In a previous study, this bacterium showed significant antagonistic activity against pathogenic bacteria in dogs [20]. In particular, the *L. animalis* SWLA-1 strain, which has strong antibacterial activity compared to other *L. animalis* strains, had superior inhibitory effects against several MDR pathogenic bacteria in vitro when compared to classic antibiotics or other probiotic bacteria [21]. Since the crude cell-free supernatant (CFS) of this bacterium has also shown antimicrobial effects against MDR bacteria, we investigated its antibacterial characteristics against MDR *S. pseudintermedius* in an in vivo experiment.

Although numerous animal models employing *Staphylococcus aureus* have been established to investigate infectious osteomyelitis, no animal model employing *S. pseudintermedius* has been reported [22,23,24]. As *S. pseudintermedius* is the primary causative agent of infectious osteomyelitis in animals, a novel animal model was established to evaluate the antibacterial activity of *L. animalis* SWLA-1-derived CFS and other treatments against MDR *S. pseudintermedius*. The establishment of an animal model utilizing *S. pseudintermedius* is important to facilitate research and reflect real-world clinical scenarios.

In this study, we assessed the inhibitory activity of concentrated *L. animalis* SWLA-1-derived CFS against MDR *S. pseudintermedius* in a novel animal model, and quantitatively compared its anti-staphylococcal activity with that of antibiotics or controls in live animals. To demonstrate that this compound is non-toxic to live animal tissues and organs, its safety was evaluated using osteoblasts and myoblasts in vitro.

## 2. Results

### 2.1. Antimicrobial Activity of L. animalis SWLA-1 and CFS against MDR Indicator Bacteria

**Figure 1:**
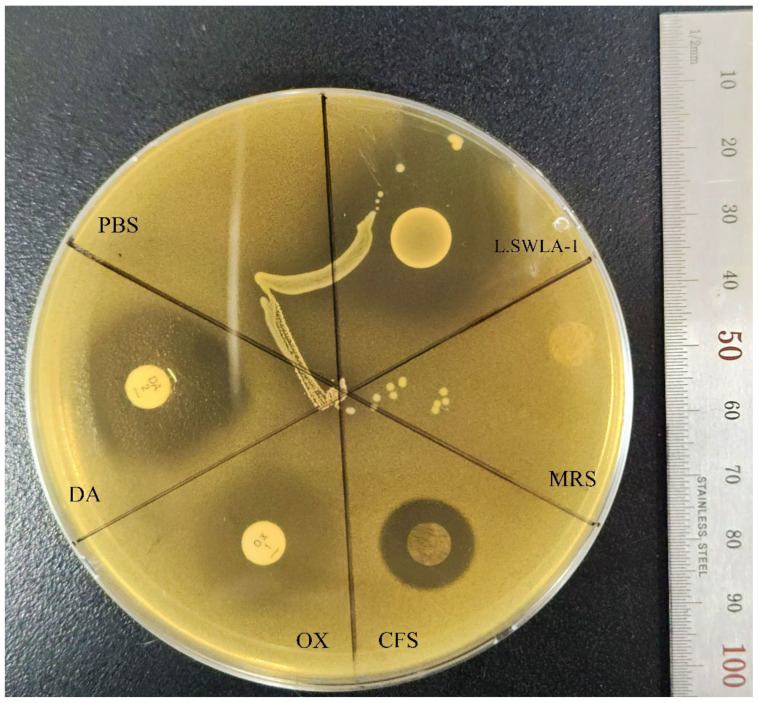
Agar spot assay with *Lactobacillus animalis* SWLA-1, concentrated CFS, and antibiotic disks against *Staphylococcus pseudintermedius* KUVM1701GC. The sections are divided as: PBS, phosphate buffered saline; DA, clindamycin (2 µg/disk); OX, oxacillin (1 µg/disk); CFS, 10-fold concentrated CFS derived from *L. animalis* SWLA-1 (118.82 ± 3.27 mg/mL); MRS, 10-fold concentrated sterile Man Rogosa and Sharpe broth (121.47 ± 4.76 mg/mL); L.SWLA-1, bacterial spot of *L. animalis* SWLA-1. The inhibition halos (mm) were observed as: PBS = 0, DA = 22.67 ± 0.58, OX = 0, CFS = 13.33 ± 0.58, MRS = 0, L.SWLA-1 = 26.67 ± 1.15. The numerical data are presented as means ± standard deviation of the experiments performed independently in triplicate.

The antimicrobial susceptibility profile of *S. pseudintermedius* KUVM1701GC was determined using the microdilution method (Supplementary Materials Table S1). This pathogenic bacterium was resistant to 11 antibiotics (ciprofloxacin, cefotaxime, ampicillin, oxacillin, ceftriaxone, tetracycline, chloramphenicol, gentamicin, azithromycin, trimethoprim/sulfamethoxazole, and ceftazidime) and susceptible to 3 antibiotics (clindamycin, imipenem, and amikacin). The growth of this methicillin-resistant and MDR *S. pseudintermedius* was more inhibited by clindamycin, a bacterial spot of *L. animalis* SWLA-1, and concentrated *L. animalis* SWLA-1-derived CFS when compared to oxacillin or concentrated Man Rogosa Sharpe (MRS; Figure 1). The minimum concentration of CFS that could inhibit the growth of the indicator bacteria on the MRS agar plate was determined to be ten-fold in this experiment, whereas no inhibitory effect was observed with equally concentrated MRS broth or oxacillin.

### 2.2. Cytotoxic Effect on Osteoblast Cell and Myoblast Cell

**Figure 2:**
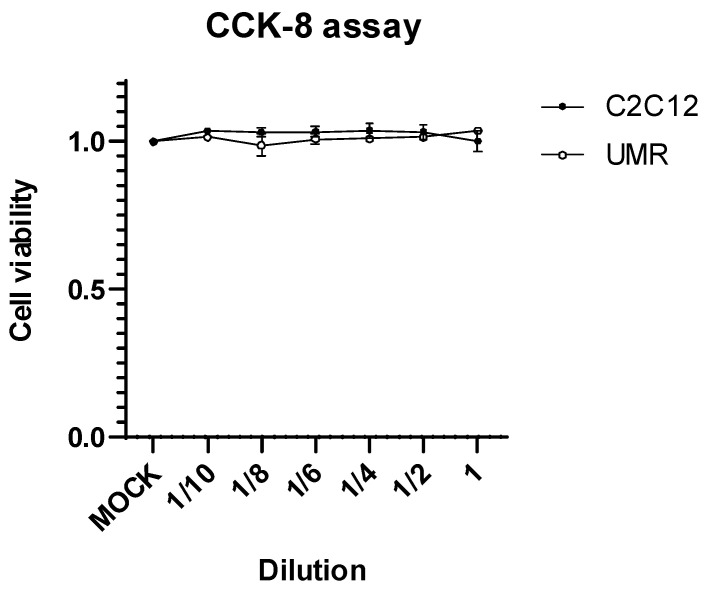
Cell viability results of C2C12 and UMR cells at 72 h post-treatment with concentrated *L. animalis* SWLA-1-derived cell-free supernatant (118.82 ± 3.27 mg/mL) when compared to MOCK-treated cells, at the indicated two-fold serial dilutions. Data are presented as means ± standard deviation of three independent experiments. CCK-8, Cell Counting Kit-8; C2C12, murine myoblast; UMR, rat osteoblast; MOCK, PBS treated mocking group.

The toxicity of concentrated *L. animalis* SWLA-1-derived CFS at the cellular level was assessed to evaluate its potential cytotoxicity in C2C12 and UMR-106 cells (derived from murine muscle and bone tissues, respectively). The results of the CCK-8 assay clearly indicated that the CFS was non-toxic, even upon exposure to the undiluted crude supernatant (Figure 2). Consequently, these findings underscore its suitability for further efficacy assessments in mammalian models.

### 2.3. Antimicrobial Activity of CFS Derived from L. animalis SWLA-1 in Novel Rat Model

**Figure 3:**
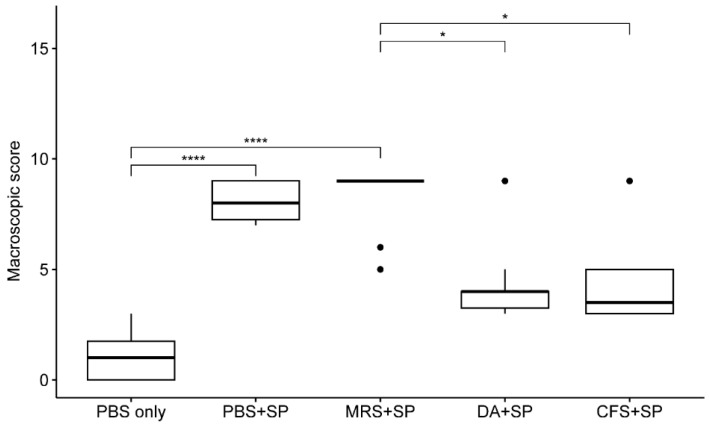
The mean differences between macroscopic scores of each experimental group. The macroscopic scores were analyzed using a Kruskal–Wallis test followed by Dunn’s test for post-hoc testing for all groups. The experimental groups are as follows: PBS only, treated with sterile PBS; PBS + SP, treated with sterile PBS after bacterial inoculation; MRS + SP, treated with 20-fold concentrated sterile MRS broth after bacterial inoculation; DA + SP, treated with clindamycin (11 mg/kg) after bacterial inoculation; CFS + SP, treated with 20-fold concentrated *L. animalis* SWLA-1-derived CFS after bacterial inoculation. Significant differences between groups are denoted by asterisks (*: *p* < 0.05, ****: *p* < 0.0001). PBS, Phosphate buffered saline; SP, *Staphylococcus pseudintermedius*; MRS, Man Rogosa and Sharpe broth; DA, clindamycin; CFS, cell-free supernatant. The outliers were present by the bullets in the figure. The mean of observed data in each experimental group was present by the horizontal bold line in the figure.

**Figure 4:**
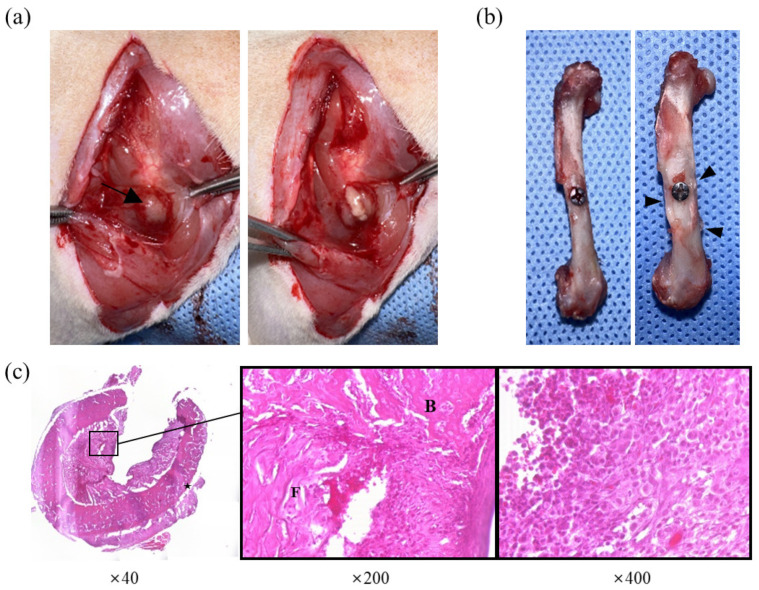
Observed lesions of group treated with PBS after staphylococcal injection. (**a**) Gross lesion at the surgical site. Note the abscess formation (arrow) above the femur and purulent exudate erupting from the capsule. (**b**) Macroscopic cortical changes in femur. Note the thickened cortex around the screw (arrowhead) compared to the femur without macroscopic changes (left). (**c**) Histological evaluation of osteomyelitis. Enlargement of cortical bone (star) can be observed, and the bone marrow cavity was filled with fibrinous exudate (F), new bone (B), and inflammatory infiltrating cells (right).

The results of the gross lesion evaluation are shown in Figure 3. The macroscopic scores of the groups treated with PBS or 20-fold concentrated MRS broth (239.32 ± 4.27 mg/mL) were significantly higher than those of the *S. pseudintermedius* native group (*p* < 0.0001) after staphylococcal injection. The scores of the groups treated with clindamycin (11 mg/kg) or 20-fold concentrated *L. animalis* SWLA-1-derived CFS (232.96 ± 5.23 mg/mL) were not significantly different from those of the negative control (*p* > 0.05). The macroscopic score of the group treated with PBS after staphylococcal injection showed no significant difference compared to the groups treated with clindamycin (11 mg/kg) or 20-fold concentrated *L. animalis* SWLA-1-derived CFS (*p* > 0.05). The group treated with MRS broth had significantly higher scores than the groups treated with clindamycin or *L. animalis* SWLA-1-derived CFS (*p* < 0.01). Specific gross lesions are shown in Figure 4a,b.

**Figure 5:**
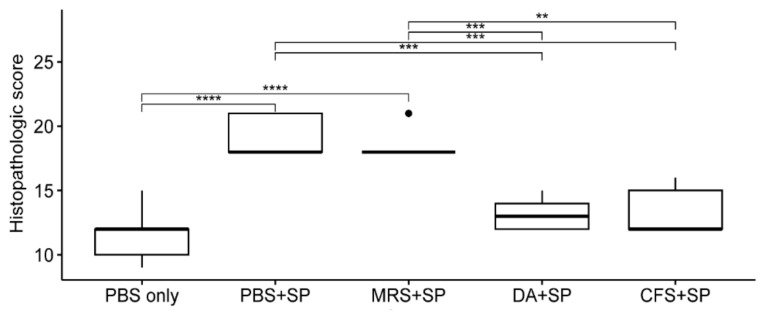
The mean differences between histopathologic scores of each experimental group. The histopathologic scores were analyzed using one-way analysis of variance (ANOVA) followed by Tukey’s test for post-hoc testing for all groups. The experimental groups are as follows: PBS only, treated with sterile PBS; PBS + SP, treated with sterile PBS after bacterial inoculation; MRS + SP, treated with 20-fold concentrated sterile MRS broth after bacterial inoculation; DA + SP, treated with clindamycin (11 mg/kg) after bacterial inoculation; CFS + SP, treated with 20-fold concentrated *L. animalis* SWLA-1-derived CFS after bacterial inoculation. Significant differences are denoted by asterisks (**: *p* < 0.01, ***: *p* < 0.001, ****: *p* < 0.0001). PBS, Phosphate buffered saline; SP, *Staphylococcus pseudintermedius*; MRS, Man Rogosa and Sharpe broth; DA, clindamycin; CFS, cell-free supernatant. The outliers were present by the bullets in the figure. The mean of observed data in each experimental group was present by the horizontal bold line in the figure.

**Figure 6:**
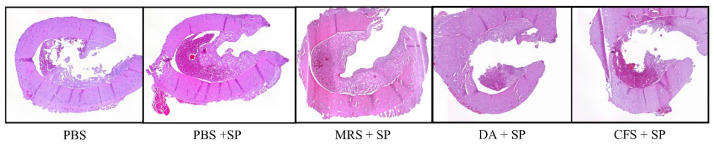
Histological comparison between groups (×40). Groups treated with clindamycin or CFS showed milder microscopic changes (similar severity as group who did not receive staphylococcal injection) when compared to groups treated with PBS or MRS broth. PBS, Phsophate buffered saline; SP, *Staphylococcus pseudintermedius*; MRS, Man Rogosa and Sharpe broth; DA, clindamycin; CFS, cell-free supernatant.

The results of the histopathological evaluation are shown in Figure 5. The histopathological scores of the groups treated with PBS or 20-fold concentrated MRS broth were significantly higher than the *S. pseudintermedius* negative group after staphylococcal injection (*p* < 0.0001). The group treated with clindamycin (11 mg/kg) had significantly lower scores than the groups treated with PBS or concentrated MRS broth (*p* < 0.001). The score of the group treated with concentrated CFS was significantly lower than those of the groups treated with PBS (*p* < 0.001) or concentrated MRS broth (*p* < 0.01). Specific osteomyelitis-related histopathological findings are shown in Figure 4c. An overview of the transverse section of the screw-implanted site in each group is shown in Figure 6.

**Figure 7:**
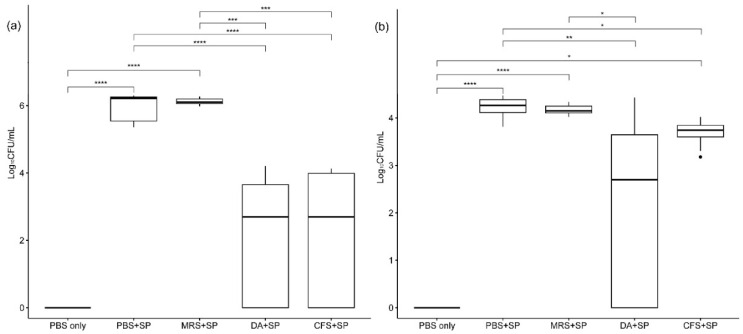
The mean differences in re-isolated indicator bacterial colony counts (log_10_ CFU/mL) from femoral bone marrow contents and screws used in animals between the experimental groups. Section (**a**) shows the colony counts from femoral bone marrow, and section (**b**) shows colony counts from suspensions made using screws from animals. The CFU values of all groups were analyzed using a Kruskal–Wallis test followed by Dunn’s test for post-hoc testing. The experimental groups are as follows: PBS only, treated with sterile PBS; PBS + SP, treated with sterile PBS after bacterial inoculation; MRS + SP, treated with 20-fold concentrated sterile MRS broth after bacterial inoculation; DA + SP, treated with clindamycin (11 mg/kg) after bacterial inoculation; CFS + SP, treated with 20-fold concentrated *L. animalis* SWLA-1-derived CFS after bacterial inoculation. Significant differences are denoted by asterisks (*: *p* < 0.05, **: *p* < 0.01, ***: *p* < 0.001, ****: *p* < 0.0001). PBS, Phosphate buffered saline; SP, *Staphylococcus pseudintermedius*; MRS, Man Rogosa and Sharpe broth; DA, clindamycin; CFS, cell-free supernatant. The outliers were present by the bullets in the figure. The mean of observed data in each experimental group was present by the horizontal bold line in the figure.

The re-isolated indicator bacterial colony counts are shown in Figure 7. The viable *S. pseudintermedius* KUVM1701GC colony count varied according to the treatment type. In the femoral bone marrow contents of rats, the colony counts of the groups treated with PBS or 20-fold concentrated MRS broth were significantly higher than those of the *S. pseudintermedius* negative group (*p* < 0.001) after staphylococcal infection. In comparison, the groups treated with clindamycin (11 mg/kg) or 20-fold concentrated *L. animalis* SWLA-1-derived CFS showed no significant difference in colony counts when compared with the negative group (*p* > 0.05). No significant difference between the anti-staphylococcal activity of clindamycin and concentrated CFS was observed based on the re-isolated *S. pseudintermedius* colony count results. Both inhibited the growth of indicator bacteria more than the other treatments (*p* < 0.01). Based on the colony counts in the surgical screw suspensions, the indicator bacteria grew significantly more in the groups treated with PBS or 20-fold concentrated MRS broth compared with those treated with PBS only, which were *S. pseudintermedius* negative (*p* < 0.001). The group treated with clindamycin after bacterial infection had significantly lower bacterial colony counts than other groups infected with *S. pseudintermedius*, except for the group treated with concentrated CFS (*p* < 0.05). The group treated with concentrated CFS also had significantly different results from the PBS-treated group after bacterial infection (*p* < 0.05), as well as in the femoral bone marrow. However, a significant difference between this group and the negative group was also observed based on the colony counts in the surgical screw suspensions (*p* < 0.05). No significant differences were observed between the groups treated with concentrated CFS and MRS based on their surgical screw suspensions (*p* > 0.05), although the mean colony-forming unit (CFU) values of the group treated with concentrated MRS were higher than those of the group treated with concentrated CFS.

## 3. Discussion

Acute osteomyelitis is considered a critical orthopedic disease in veterinary medicine because it can lead to serious medical and financial consequences if not detected and treated properly in the early phase. Osteomyelitis is diagnosed via clinical signs, radiography, and microbiological cultures [25,26]. It is relatively difficult to confirm a diagnosis of osteomyelitis in the early stages. Acute and chronic osteomyelitis are somewhat differentiated based on the duration of progression, with the acute phase being characterized by soft-tissue swelling within a day or clinical signs within a few days. However, a specific time point has been defined to precisely differentiate between these two conditions [7,27,28]. Radiological findings such as a lamellated periosteal reaction may indicate acute osteomyelitis, but clear differentiation becomes difficult in cases of mild severity or interference from implants [27,28]. Although several studies have attempted to define acute osteomyelitis on the basis of histology, variations may exist among patients and/or lesions [27,29]. Considering these characteristics, it is important to recognize that every patient with infectious osteomyelitis can potentially develop severe complications. Recent research suggests that features of chronic osteomyelitis can develop within two weeks, and some rats in the present study showed relatively severe signs. These findings highlight the importance of early treatment and prophylaxis in acute osteomyelitis [27].

*Staphylococci* are the main bacteria responsible for osteomyelitis, with *S. aureus* being predominant in humans and *S. pseudintermedius* in animals [5,6]. Because osteomyelitis is an important issue in human medicine, numerous studies using animal models infected with *S. aureus* have been conducted [30]. Although bacterial osteomyelitis is also a significant concern in veterinary orthopedic surgery, it is difficult to find studies reporting on animal models of osteomyelitis using *S. pseudintermedius*. To our knowledge, this is the first study to develop an animal model of infectious osteomyelitis using *S. pseudintermedius*. To simulate real-world clinical scenarios observed in veterinary orthopedics, a titanium screw was used to cover the defect which served as the inoculation site for the bacteria. Previous studies utilizing *S. aureus* for in vivo osteomyelitis models used 10^2^ and 10^9^ CFU in the inoculum. We injected 9 × 10^4^ CFU of *S. pseudintermedius* to induce acute osteomyelitis in this study [31,32,33].

With the emergence of MDR bacteria and resistance to classical antibiotics increasing worldwide, the need for novel antimicrobial compounds has also increased [34]. Furthermore, alternatives to classic antibiotics are urgently needed to secure the efficacy of these critically important antibiotics for human and veterinary medicine [10,11]. In veterinary medicine, methicillin-resistant *S. pseudintermedius* (MRSP) and MDR *S. pseudintermedius* are the major pathogens responsible for various challenging bacterial infections in animals [35,36]. We evaluated the anti-staphylococcal activity of *Ligilactobacillus animalis* SWLA-1, which has versatile antimicrobial activity against both Gram-positive and Gram-negative MDR bacteria [21], against *S. pseudintermedius* KUVM1701GC in a novel animal model. Since the crude CFS derived from these bacteria also showed antimicrobial activity against MDR bacteria after 0.45 µm filtration, we evaluated its antagonistic activity against MDR *S. pseudintermedius* in live animals. In this study, the potential of CFS as a novel alternative to classic antibiotics against MDR *S. pseudintermedius* was evaluated by comparing its antimicrobial activity with that of other treatments. The antimicrobial activity and cytotoxicity of the concentrated *L. animalis* SWLA-1-derived CFS were evaluated via in vitro experiments in this study. The antimicrobial activity of *L. animalis* SWLA-1 and its concentrated CFS was determined using a spot agar assay as previously described [21], and compared with that of clindamycin, oxacillin, and concentrated sterile MRS broth. The inhibitory characteristics of *L. animalis* SWLA-1 and its concentrated CFS against *S. pseudintermedius* KUVM1701GC were similar to those of clindamycin (and superior to those of oxacillin or concentrated MRS), which the indicator bacteria are susceptible to. This suggests that the antimicrobial efficacy is attributed to novel antibacterial compounds in the supernatant of *L. animalis* SWLA-1, regardless of the ingredients in the MRS broth. The concentrated CFS showed no significant cytotoxic effects to C2C12 and UMR-106 cells. Considering that most lactobacilli probiotics are of GRAS (generally recognized as safe) grade according to the USA Food and Drug Administration (FDA) guidelines, and *L. animalis* ATCC strains have been tested for safety in animals in a previous study by the European Food Safety Authority [37], we believe that *L. animalis* SWLA-1-derived CFS can be effective and safe in live animals.

The in vivo osteomyelitis experiment was conducted in three parts: gross lesion evaluation, histopathological evaluation, and microbiological evaluation, based on the parameters used in previous studies [27,31,38,39]. In this study, we focused on evaluating lesions and osteomyelitis specimens from animals at an early stage. Gross lesion evaluation findings included abscess formation and cortical changes in the femur around the bacterial injection site. The *Staphylococcus*-positive groups treated with PBS or concentrated MRS showed significantly higher macroscopic scores than the *Staphylococcus*-negative group (*p* < 0.001). The scores of the groups treated with clindamycin or concentrated CFS were not significantly different from that of the *Staphylococcus*-negative group but were significantly lower than that of the group treated with MRS (*p* < 0.05). Additionally, there were no significant differences between the groups treated with clindamycin, CFS, or PBS. Gross evaluation primarily examines the morphological features of the lesion. In the case of bones, such features can also be detected through radiography. However, during the acute phase of osteomyelitis, morphological changes such as periosteal reaction, osteolysis, and sequestrum formation are often not clearly detectable [7,31,40]. Therefore, histopathological and microbiological evaluations were used in conjunction with macroscopic findings for gross lesion evaluations in this study.

The scores determined by the histopathological findings varied among the experimental groups. The groups that received clindamycin or concentrated CFS had significantly lower scores than the groups treated with PBS or concentrated MRS (*p* < 0.01). Fibrinous exudates with infiltrating inflammatory cells, new bone formation in the bone marrow cavity, and cortical bone enlargement were observed in all groups. However, the findings in the *Staphylococcus*-negative group are considered to be induced by the traumatic nature of screw implantation rather than infection. The groups treated with clindamycin or concentrated CFS showed no significant differences compared to the *Staphylococcus*-negative group, suggesting that concentrated CFS has antimicrobial activity similar to clindamycin treatment. Similar results were observed in the microbial evaluation. Based on the bacterial colony counts, the indicator bacterial growth was significantly inhibited in the group treated with concentrated CFS compared to the group treated with clindamycin (*p* < 0.001). Thus, the antimicrobial compounds in *L. animalis* SWLA-1-derived CFS may inhibit pathogenic MDR bacteria in the bone marrow which are non-susceptible to clindamycin, an antibiotic that is usually used to treat staphylococcal infection. However, the bacterial colony count results in the suspensions of the secreted specimens were marginally different. The CFU was significantly lower in the group treated with CFS than that of the *Staphylococcus*-positive PBS-treated group (*p* < 0.05), but a significant difference was also observed when compared with the CFU data of the *Staphylococcus*-negative group (*p* < 0.05). This result may be explained by biofilm formation or other factors related to screw application rather than the characteristics of the CFS itself. Introducing methods to promote resistance to bacterial biofilms and long-lasting materials which constantly secrete antimicrobial compounds should be considered in future studies to improve the outcomes of using surgical implants.

Various methods involving direct antibiotic administration at the surgical site exist for preventing osteomyelitis. Antibiotic-loaded polymethylmethacrylate (PMMA) bone cements and beads have been widely studied [41,42,43]. However, bone cement may not be suitable for every fracture, and its non-biodegradable nature raises uncertainty regarding how long antibiotics remain in the body. One study reported that released antibiotics were detected even 5.5 years after surgery, increasing the potential risk of antibiotic resistance [44]. Furthermore, cement that no longer releases antibiotics may serve as a surface for biofilms, and cement-removal surgery can lead to other perioperative infections [27,45]. Another method used is implant coating [2,4,46]. Although studies on antibiotic orthopedic implant coatings have shown promising results, their application in clinical practice is limited owing to issues such as cytotoxicity, immune responses, and economic costs associated with the coating materials. Moreover, antibiotic coatings inevitably promote antibiotic resistance [2,46,47]. In this context, antimicrobial substances derived from probiotics, such as bacteriocins and other antimicrobial molecules, are promising alternatives to classic antibiotics. These compounds are amenable to bioengineering and are less prone to inducing antibiotic resistance [48]. Additionally, collagen-derived materials, which were used in this study, are biodegradable materials that are commonly used in surgery as drug-delivery systems. Antibiotic-containing collagen sponges can achieve high drug concentrations and minimize the risk of antibiotic resistance owing to their limited duration of action [49]. Furthermore, collagen products can be cut to the desired size, allowing for more flexible use at various surgical sites. In this study, the collagen patch served as an effective delivery system to enable antimicrobial activity at the staphylococcal infection sites.

Based on our experimental data, canine infection models for investigating various types of infectious osteomyelitis should be established in future studies. These models may contribute to the discovery and development of novel therapeutic approaches which reflect clinical needs. In addition, genome analysis should be performed to identify the genes or sequences encoding novel antimicrobial molecules in the complete genome of *L. animalis* SWLA-1. Peptide and chemical analyses of the *L. animalis* SWLA-1-derived CFS should also be conducted to identify and purify its active antimicrobial molecules. These studies will inform the discovery and development of novel antimicrobial agents and contribute to the development of critically important antibiotics.

## 4. Materials and Methods

### 4.1. Preparation of Bacterial Strains and Crude CFS

A frozen pure culture of *L. animalis* SWLA-1 was thawed and placed on Difco™ MRS agar (BD Biosciences, Sparks, MD, USA). The plates were then incubated at 37 °C for 24 h. Five single *L. animalis* SWLA-1 colonies were inoculated into 10 mL of MRS broth and incubated overnight. This bacterial culture was used in the agar spot assay and for CFS preparation.

*L. animalis* SWLA-1 pre-cultured overnight in MRS broth (≈1.2 × 10^9^ CFU/mL) was collected and centrifuged at 10,000× *g* and 4 °C for 30 min (Legend X1R; Thermo Fisher Scientific, Waltham, MA, USA) to obtain the crude CFS. Since the antimicrobial activity of this CFS was relatively higher after concentration based on a previous study [21], it was concentrated using the trichloroacetic acid (TCA) protein precipitation method. Briefly, 10 mL of TCA solution (Sigma-Aldrich, St. Louis, MO, USA) was added to 40 mL of crude *L. animalis* SWLA-1-derived CFS. The samples were incubated for 1 h at 4 °C for precipitation. The sample was centrifuged at 14,000× *g* and 4 °C for 10 min. The supernatant was then removed, leaving the protein pellet intact. This pellet was washed with 2 mL of chilled acetone and centrifuged at 14,000× *g* and 4 °C for 10 min. The washing step was repeated once more, and the pellet was dried in a 95 °C heat block to evaporate off any residual acetone. The pure protein pellet was re-dissolved in 50 µL of dimethyl sulfoxide (Sigma-Aldrich, St. Louis, MO, USA) and diluted in 950 µL of Milli-Q water. Sterile MRS broth was also concentrated with TCA solution in the same manner and used for antimicrobial activity comparisons. The protein concentrations in the concentrated MRS and CFS were quantified using a bicinchoninic acid (BCA) protein assay (Pierce™ BCA Protein Assay Kits, Thermo Fisher, Rockford, IL, USA), and compared with those in normal MRS broth and crude CFS.

A multidrug-resistant (MDR) *Staphylococcus pseudintermedius* strain, *S. pseudintermedius* KUVM1701GC (isolated from a clinical sample provided by Konkuk University Veterinary Medical Teaching Hospital (KUVMTH)), was used as an MDR indicator bacterium in this study. A frozen pure culture of *S. pseudintermedius* KUVM1701GC was thawed and placed on sheep’s blood agar. The agar plates were then incubated at 37 °C for 24 h. Five single colonies of this bacterium were inoculated into 10 mL of tryptic soy broth (Sparks, MD, USA) and incubated overnight. These bacterial cultures were used for the agar spot assay. The antimicrobial susceptibility profiles of these bacteria were evaluated using the microdilution method with 14 antimicrobial agents. Briefly, pre-cultured *S. pseudintermedius* KUVM1701GC was diluted in sterile TSB. After that, 10µL of this diluted bacterial culture (A_600_ = 0.098) was inoculated in 10 mL of BBL™ Müller–Hinton II broth (BD, Sparks, MD, USA). This bacterial inoculum was placed in a 96-well microplate which contained serially two-fold diluted antimicrobial agents. This procedure was performed according to the protocol of the Clinical Standard Laboratory Institute (CLSI).

### 4.2. Evaluation of Antimicrobial Activity against MDR Indicator Bacteria

The antimicrobial activities of *L. animalis* SWLA-1 and its concentrated CFS against *S. pseudintermedius* KUVM1701GC were evaluated on MRS plates. As the MDR indicator bacteria were susceptible to clindamycin (DA, 2 µg/disk) and resistant to oxacillin (OX, 1 µg/disk), antibiotic disks (Oxoid, Cheshire, UK) were used as references in this experiment. The pre-culture of *L. animalis* SWLA-1 (37 °C for 4 h, 200 rpm) was prepared and 3 µL of this bacterial culture (A_600_ = 0.500) was spot inoculated on a section of the MRS agar plate. The spot contained 1.5 × 10^5^ CFU. An equal volume of phosphate-buffered saline (PBS) was added to another section of the MRS agar plate. Plates containing inoculated bacterial spots were incubated at 37 °C for 24 h. The DA and OX disks were also placed on separate sections of the same plate. The concentrated MRS broth and *L. animalis* SWLA-1-derived CFS were placed on the remaining sections plate after being absorbed in a porcine-derived collagen patch (InterCollagen Guide, SigmaGraft Inc., Fullerton, CA, USA) of the same size as the antibiotic disks. Subsequently, the MDR indicator bacterial culture (~2 × 10^9^ CFU/mL) was mixed with 10 mL of soft Müller–Hinton agar (0.8%) and overlaid on the prepared MRS agar plate. After the molten overlaid agar solidified, each plate was incubated at 37 °C for 24 h. This experiment was performed independently in triplicates.

### 4.3. Cytotoxicity Assay

The cytotoxicity of concentrated *L. animalis* SWLA-1-derived CFS was determined using osteoblast and myoblast cells to determine whether it was suitable for use in animal models of acute osteomyelitis. C2C12 (ATCC No. CRL-1772™) and UMR-106 (ATCC No. CRL-1661™) cells were acquired from the American Type Culture Collection (Rockville, MD, USA) and cultured in Dulbecco’s modified Eagle’s medium (DMEM) supplemented with 8% heat-inactivated fetal bovine serum (FBS, Gibco, Grand Island, NY, USA) at 37 °C in a 5% CO_2_ atmosphere. The cytotoxic effects of the CFS on C2C12 and UMR-106 cells were evaluated using a CCK-8 assay. C2C12 and UMR-106 cells were seeded at a density of 1 × 10^4^ cells/well in 96-well plates. After 24 h, the cells were treated with 2.5 μL of serial concentrations (original concentration, 2-, 4-, 6-, 8-, or 10-fold dilution) of concentrated *L. animalis* SWLA-1-derived CFS or MRS medium (MOCK control group) in a combination with 97.5 μL of DMEM for 72 h. After the supernatant was removed, 10% CCK-8 reagent (96992, Sigma-Aldrich, St. Louis, MO, USA) in fresh serum-free media was added to the cells, which were then incubated in a 5% CO_2_ atmosphere at 37 °C. After 3 h, a spectrophotometer was used to measure the absorbance of the viable cells at 450 nm. Cytotoxicity was analyzed by comparing the viability with that of mock-treated cells, which were considered as 100% viable controls. This experiment was performed independently in triplicates.

### 4.4. Establishment of Novel Acute Osteomyelitis Rat Model Using S. pseudintermedius

Fifty 14-week-old male Sprague Dawley rats (Daehan Biolink, Eumsung, Republic of Korea) were used. The rats were divided into five experimental groups (N = 10); the specific treatments for each group are shown in Table 1. The surgical procedure was performed under general anesthesia via isoflurane inhalation (IsoVet; Piramal Pharma Ltd., Mumbai, India) in a sterilized operation unit. The surgical procedure was as follows: the rat was placed in left lateral recumbency, and the skin over the right hind limb was prepared aseptically. A 2 cm skin incision was made over the craniolateral aspect of the right femur. The subcutaneous tissue and superficial fascia were bluntly dissected. The exposed fascia lata was incised and the biceps femoris was retracted caudally to expose the femoral shaft. A unicortical hole was drilled using a 0.9 mm diameter drill bit. In Group 1, right after the hole was made, a 4 mm diameter porcine-derived collagen patch (InterCollagen Guide, SigmaGraft Inc., Fullerton, CA, USA) was placed over the hole and 20 µL of PBS was applied to the patch. A titanium screw (1.2 mm in diameter and 3 mm in length; Imedicom, Gunpo, Republic of Korea) was inserted into the hole over the patch using a hand driver. In groups 2–5, after 20 µL of pre-cultured *S. pseudintermedius* KUVM701GC (A_600_ = 0.200, 4.5 × 10^6^ CFU/mL) was inoculated into the hole, a collagen patch was attached to cover the hole and 20 µL of sterile PBS (Group 2), concentrated MRS (Group 3), 1.1 mg clindamycin (Group 4), or concentrated CFS (Group 5) was absorbed in each patch, respectively. The clindamycin concentration (11 mg/kg) was determined according to the recommendations of the US FDA and a previous study on dosage in animal osteomyelitis [50,51]. To maintain the minimum effective concentration of CFS after mixing with the bone marrow contents, twenty-fold concentrated MRS and CFS were used in this experiment. This was based on the estimate that the volume of femoral bone marrow contents is approximately 20 µL. The screw was inserted using the same method as in Group 1. The incised fascia and skin were closed with 4-0 sutures (PDS, Ethicon, Norderstedt, Germany; Dafilon, B.Braun, Rubi, Spain). The animals were kept in separate cages after surgery. Animals were humanely euthanized on the 7th post-procedural day via cardiac potassium chloride injection under general anesthesia. The femur of the right hind limb was dissected and collected under sterile conditions for gross lesion, histological, and microbiological evaluations. All procedures were approved by the Institutional Animal Care and Use Committee (IACUC) of Konkuk University (approval number: KU23095).

### 4.5. Gross Lesion Evaluation

Gross lesions were evaluated using a modified version of Rissing’s classification: 0, absence of an abscess, sequestra, bone formation, or erythema; 1, minimal erythema without abscesses or new bone formation; 2, erythema with cortical changes; 3, abscess with cortical changes; 4, severe bone resorption, abscesses, and total femoral involvement [38]. All samples were scored independently by three observers. The maximum possible score was 12.

### 4.6. Bone Histopathologic Evaluation

Five femurs from each group were fixed for two days in 5% formaldehyde and decalcified using a decalcifying agent (DO818, Sigma-Aldrich, St. Louis, MO, USA). The femurs were embedded in paraffin and cut into 4 μm thick sections. The slices were stained with hematoxylin and eosin. The following parameters were evaluated to determine the severity of osteomyelitis [27,31,39]: (1) granulocyte infiltration, (2) enlarged cortical bones, (3) new bone formation, (4) fibrinous exudate, and (5) general impressions. The parameters were scored as 0 (absent), 1 (mild), 2 (moderate), or 3 (severe). All histological samples were independently scored on each parameter by three observers. The maximum possible score was 21.

### 4.7. Microbiological Evaluation

Five femur samples from each group were evaluated using bacterial re-isolation to assess the viable colony counts on *Staphylococcus*-selective media. Screws and 20 µL of bone marrow were collected from the femur samples and suspended in 10 mL of sterile PBS via vortexing. The PBS-suspended samples were serially diluted by 10-fold and 20 µL of each dilutant was placed on *Staphylococcus* No. 110 medium plates (Oxoid, Basingstoke, Hants, UK) according to the method described by Miles and Misra for viable bacterial colony counting. The plates were then incubated at 37 °C for 24 h. After incubation, the bacterial colonies were counted and the measured CFU value of each sample was logarithmically transformed (log_10_ CFU/mL) to compare the mean differences in counts between groups. The experiments were performed independently in triplicate.

### 4.8. Statistical Analyses

The quantitative data of the experimental results in this study were evaluated for normality using the Shapiro–Wilk test. Since the gross lesion and microbiological evaluation data were not normally distributed, the Kruskal–Wallis test was used to non-parametrically compare the multiple means of the experimental data from each group. This test was used to analyze any differences in the observed mean macroscopic scoring or CFU data between groups. Subsequently, post-hoc Dunn’s testing was used to compare the mean macroscopic scoring and CFU data of each group. The results of the bone histopathological evaluation were normally distributed. The means of the histopathological scoring data of each group were compared using one-way ANOVA. Post-hoc Tukey’s testing was used to compare the scores of each group. All tests were performed using the “rstatix” package in R (version 0.6.0; R Foundation for Statistical Computing, Vienna, Austria; Alboukadel Kassambara, 2020). Significance was set at an α level of 0.05.

## 5. Conclusions

The results of our study suggest that concentrated *L. animalis* SWLA-1-derived CFS significantly inhibits the growth of MDR *Staphylococcus pseudintermedius* in live animals. This compound has potential as a strategy to control bacterial infections in bone during surgery or wound-related osteomyelitis. With further purification or bioengineering, the active molecules in this CFS may be used as novel antimicrobial agents against MDR staphylococcal infections. This study is the first to investigate *Staphylococcus pseudintermedius*-induced bacterial osteomyelitis in live animals using our novel animal infection model.

## Figures and Tables

**Table 1:** Experimental group design for *Staphylococcus pseudintermedius* challenge.

Group	Number of Rats	Challenge	Treatment
Group 1	10	PBS	PBS
Group 2	10	*Staphylococcus pseudintermedius* (9 × 10^4^ CFU)	PBS
Group 3	10	*Staphylococcus pseudintermedius* (9 × 10^4^ CFU)	Sterile MRS broth
Group 4	10	*Staphylococcus pseudintermedius* (9 × 10^4^ CFU)	Clindamycin (11 mg/kg)
Group 5	10	*Staphylococcus pseudintermedius* (9 × 10^4^ CFU)	Concentrated CFS of *L. animalis* SWLA-1

Ten rats in each group were inoculated with 20 µL of pre-cultured bacterial inoculum (4.5 × 10^6^ CFU/mL) per animal, which is equivalent to 9 × 10^4^ CFU (except for animals in Group 1, which acted as *Staphylococcus*-negative controls). PBS, Phosphate buffered saline; MRS, Man Rogosa and Sharpe broth; CFS, cell-free supernatant.

## Data Availability

The data are contained in the manuscript or Supplementary Materials.

## Supplementary Materials

The following supporting information can be downloaded at https://www.mdpi.com/article/10.3390/antibiotics12091444/s1, Table S1. Antimicrobial susceptibility profile of *Staphylococcus pseudintermedius* KUVM1701GC with minimum inhibitory concentration, Supplementary File S1. The raw data of bacterial colony counts in in vivo Staphylococcus pseudintermedius infection.
