# Supplementary material for: Anti-Staphylococcal Activity of Ligilactobacillus animalis SWLA-1 and Its Supernatant against Multidrug-Resistant Staphylococcus pseudintermedius in Novel Rat Model of Acute Osteomyelitis
Source: Antibiotics (Basel). 2023 Sep 13;12(9):1444. doi: 10.3390/antibiotics12091444 (PMC10526016; doi:10.3390/antibiotics12091444)
Supplement: Supplementary file 1 [file antibiotics-12-01444-s001.zip › antibiotics-2599643-Supplementary Table S1-after proofreading.pdf]

Table S1. Antimicrobial susceptibility profile of the *Staphylococcus pseudintermedius* KUVM1701GC strain with the minimum inhibitory concentration.

Antibiotics	MIC (μg/mL)											Resistance breakpoint (μg/ml)
	≤0.125	0.25	0.5	1	2	4	8	16	32	64	128≤	
Ciprofloxacin								16				1
Clindamycin					2							4
Imipenem				1								4
Cefotaxime									32			32
Ampicillin										64		0.5
Oxacillin								16				0.5
Ceftriaxone									32			4
Tetracycline										64		1
Chloramphenicol									32			32
Gentamicin								16				16
Azithromycin									32			2
Amikacin						4						16
Trimethoprim/ Sulfamethoxazole							8/152					4/76
Ceftazidime								16				1

Minimum inhibitory concentration (MIC) values were determined according to Clinical and Laboratory Standards Institute protocols M100, vet01 (CLSI, 2019) and The European Committee on Antimicrobial Susceptibility Testing Version 11.0 (EUCAST, 2021). Red background - resistant, grey background - susceptible
